# Reconstructing the historical synthesis of mauveine from Perkin and Caro: procedure and details

**DOI:** 10.1038/s41598-017-07239-z

**Published:** 2017-07-28

**Authors:** Tânia F. G. G. Cova, Alberto A. C. C. Pais, J. Sérgio Seixas de Melo

**Affiliations:** Coimbra Chemistry Centre, Department of Chemistry, University of Coimbra, P3004-535 Coimbra, Portugal

## Abstract

Mauveine, an iconic dye, first synthesised in 1856 still has secrets to unveil. If nowadays one wanted to prepare the original Perkin’s mauveine, what would be the procedure? It will be described in this work and lies on the use of a 1:2:1 (mole) ratio of aniline, *p*-toluidine and *o*-toluidine. This was found from a comparison of a series of products synthesized from different proportions of these starting materials, with a set of historical samples of mauveine and further analysed with two unsupervised chemometrics methods.

## Introduction

**Figure 1:**
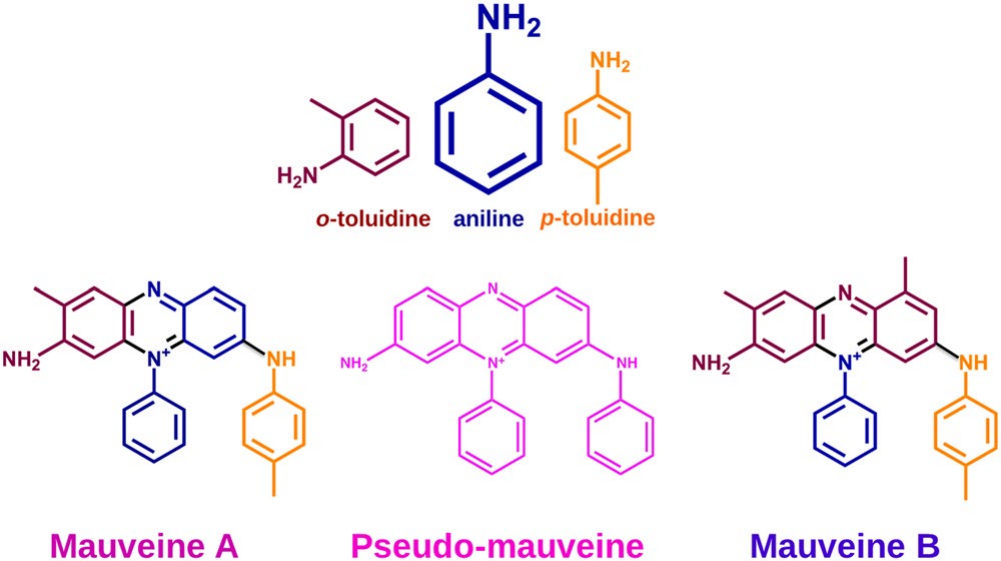
Chemical structures of aniline, *o-*toluidine and *p-*toluidine used in the synthesis of mauveine. The combination of these starting materials leading to mauveine A and B, two of the major components in mauveine samples, is depicted in different colours. Pseudo-mauveine consisting of a non-methylated structure is also presented.

Mauveine is an iconic mixture of compounds, and a landmark in the history of organic synthesis. It was the first commercially successful synthetic organic dye and was obtained accidentally by William H. Perkin in 1856 while attempting to synthesize the antimalarial drug quinine. The main feature that attracted so much attention was the noticeable purple colour and the fact that it fixed persistently to silk. Initially branded as Tyrian Purple, associated to the rare colour of the ancient dye obtained from the Mediterranean sea snail Murex Brandaris, it was later recognized as mauveine (from the French word Mauve for mallow flower) a much more fashionable word. The mystic behind this dye is not only a story of serendipity and entrepreneur spirit but also what is considered to have been the first science-based industry. Indeed, it is rightfully associated to the genesis of the dye industry, featured by the use of synthetic organic dyes over natural colorants^1–6^. The golden period of mauveine as a dye was short and almost out-dated by the mid-1860s. The only exception was found in its use for printing stamps, where in the mid to late 1860s there was some greater control over the composition of the starting materials (hydrocarbons), even then almost certainly based on boiling points from tar fractions^7^. The original composition of mauveine was previously identified from samples of museum collections and can still be found in UK Victorian 6d postage stamps (from 1867 to 1880 period)^2, 8^. Historical samples of mauveine consist of a blend of more than thirteen different methyl derivatives (C_24_ to C_28_) of 7-amino-5-phenyl-3-(phenylamino)phenazin-5-ium compounds^2^, differing in the number of methyl groups, which range from none (pseudo-mauveine) to four (mauveine D)^2^. These derivatives present absorption maxima in the 540-550 nm range, leading to mauveine’s purple color^9^. The major components include mauveine A and mauveine B (see structure in Fig. 1).

Besides William Perkin, Heinrich Caro, that worked at Roberts, Dale & Co. in Manchester, also produced mauveine with a different recipe and final composition^8, 10^. In the presence of pseudo-mauveine, the non-methylated derivative, mauveines A and B can be used as tracers for the original recipes of Perkin’s and Caro’s Mauveine^6, 10^. A specific fingerprint was revealed in which mauveines A or B were dominant, and in which mauveines B2 and C_25_ were important components for exploring the original synthesis. However, the synthetic procedure leading to this particular mixture of compounds is yet to be established. The patent letter by Perkin for the “Invention of Producing a New Coloring Matter for Dyeing with a Lilac or Purple Color Stuffs of Silk, Cotton, Wool, or other Materials” gives limited information on the synthesis of mauveine: “Equivalent proportions of sulphate of aniline and bichromate of potassium are to be dissolved in separate portions of hot water, and, when dissolved, they are to be mixed and stirred, which causes a black precipitate to form”^11^. It is known that Perkin initially started with (impure) allyltoluidine; yet, as latter mentioned in 1956, chemists have experienced difficulty in preparing a small scale mauveine equal in quality to that of Perkin & Sons manufactured a century before.^12^ A synthesis from aniline and toluidine isomers was latter formulated, mirroring presumably the Perkin synthesis^12, 13^. This is illustrated in Fig. 1, together with the structures (in color) of aniline, *o*-toluidine and *p*-toluidine as starting materials showing that the combination of these three different starting compounds leads to mauveine A and B. The pseudo-mauveine structure is also presented.

## Results and Discussion

Recently^8^, a very high percentage of pseudo-mauveine (C_24_) was found in Caro’s mauveine, whereas the C_27_ derivative, mauveine B, was the major compound in Perkin’s mauveine. This was discovered from a comparison of the chemical profile of the dyes used in a set of lilac postage stamps of the Victorian period (established based on HPLC-DAD-MS analysis^8^) with historical samples from both Perkin’s and Caro’s mauveine, both found to be used in the manufacture of those postage stamps^8^. In order to validate these results, an interpretation that involves taking the simultaneous account of several mauveine chromophores is proposed, facilitating the graphical visualization of different chemical profiles. This was carried out resorting to hierarchical cluster analysis (HCA) and principal component analysis (PCA), as described in the Methods section. In a first approach, both stamps and historical samples were characterized on the basis of the major tracers related to the mauveine structures C_24_ (pseudo-mauveine), C_25_, C_26_ (mauveine A), C_27_ (mauveine B), C_27_B3B4, C_28_ (mauveine C), C_29_ (mauveine D) and carminic acid (obtained from the cochineal extract). This was performed to confirm the correct source of the mauveine (Perkin’s or Caro’s mauveine) present in the extracts of the UK Victorian postage stamps. Specifically, the data set contains information on 10 selected postage stamps (labelled according to the Stanley Gibbons catalogue number, SG) and 8 historical samples obtained from the Science Museum, in London, UK, (denoted as ScM1–4), the Museum of Science and Industry, in Manchester, UK, (denoted as MS1IM1 and MS1M2), the Chandler Museum (CM), in Arizona, USA, and a synthesised mauveine by Caro from the Deutsche Museum (DMC), in Munich, Germany, (for details see ref. 8). As previously reported^8^, due to the lower signal of the mauveine chromophores in the extracts of Penny Lilac stamps SG170 to SG174, these were considered as a single object in the analysis, reducing the number of samples to 14. The chemical profile obtained from the postage stamps was established according to the prevalence of mauveine compounds present the historical mauveine samples, and attributed on the basis of their m/z and respective fragmentation patterns^8^. Figure S1 in the Electronic Supporting Information (ESI) presents a dendrogram representing the similarity, in terms of the m/z information, between the UK Victorian postage stamps and the historical samples. This particular data structure displays five groups of samples in which it is apparent that the distribution of those groups reflects the C_26_/C_27_ ratio, as found in previous studies (see ref. 8). For example, the group containing the SG106 stamp and the MS1M2 and DMC samples (see Fig. S1) includes a C_26_/C_27_ ratio from 3.5 to 9.4. The SG170-4 samples present a very distinct chemical profile, with no C_26_ or C_27_ mauveine structures. As seen from Fig. S2 in the ESI, the relative positioning of the samples can be further interpreted resorting to a biplot representation. It is worth noting that both the mauveine chromophores and samples are depicted on the same diagram, which allows interpreting the mauveine tracers while inspecting samples’ position. The mauveine chromophores are depicted into a so-called correlation circle, in which the angle formed by any two chromophores, represented here as vectors, reflects their actual pairwise correlation.

In Fig. S2, the points representing the stamps and the historical samples are mainly distributed along the first component, PC1, which reflects an increase of C_24_ and C_25ab_ (two isomers) fractions, from left to right, being strongly present in the SG106 stamp and in the MS1M2 and DMC samples, and an increase of C_26_, C_27_, C_27_ B3 and B4, C_28_ and C_29_ (mauveine D) fractions, from right to left. The latter structures are dominant in ScM1-4, CM, MS1M1 samples and in the SG108, SG109 and SG109_2 stamps. The second component is mostly related with the carminic acid (Coch), C_25ab_, C_24_ and C_26_ fractions. A strong relationship between C_28_ and C_29_, C_27_ and C_27_ B3 and B4 and a weak correlation between Cochineal and C_24_, C_25_, C_28_ and C_29_ are also extracted from the angles between the vectors corresponding to these fractions. The correlation between Cochineal and each of the other fractions is strongly negative. As the C_26_ and C_27_ fractions have the same loadings in this first component, an additional variable can be naturally estimated reflecting the C_26_/C_27_ ratio and can be projected on the second component along which the samples are ranked. The C_26_/C_27_ ratio, considered now as a response variable, was not included in the analysis. It should be noted that high values of C_26_/C_27_ reflect the process used by Caro^8^. From all the analyzed samples Mauveines B are dominant with no signals (or only residual traces) of pseudomaveine on ScM1, ScM2, ScM3, ScM4, MS1M1 and CM); only for MS1M2 and DCM pseudomauveine is dominant and show high values of the C_26_/C_27_ ratio. The first samples point out for a synthetic recipe followed by Perkin whereas the latter two, MS1M2 and DMC, point out for Caro’s synthetic procedure. This suggests that there are significant dissimilarities in the distribution of mauveine compounds amongst the samples included in the groups, defined in Fig. S1. The main feature for Caro’s mauveine is the presence of a high quantity of pseudo-mauveine (C_24_), in contrast to Perkin’s mauveine, which is characterized by the absence of C_24_ and a high C_26_/C_27_ (mauveine A/mauveine B) ratio. Figure S2 indicates that SG107, SG109_2, SG108 and SG109 stamps were dyed with mauveine obtained from the process used by Perkin, whereas SG106 was dyed from Caro’s procedure. Penny Lilacs SG170–4 stands out from the set, once it contains only carminic acid (Coch). These stamps contain the cochineal extract (carminic acid), and a null C_26_/C_27_ ratio. The SG106 extract stands out with the highest C_26_/C_27_ ratio, and C_24_ and C_25_ fractions, and do not present C_28_, C_29_ and carminic acid, thus relating to the use of Caro’s synthetic procedure. SG108 also stands out with the highest values of C_29_ and C_28_ fractions, followed by SG109, SG107 and SG109_2. The latter three have high fractions of C_26_ and C_27_ B3 and B4 and similar fractions of C_24_ and C_25_. This suggests that these stamps were dyed with mauveine from Perkin’s recipe.

It is worth stressing that the synthesis of mauveine requires the use of impure aniline (with o- and p-toluidine). Although the relevance of this historical dye, and the fact that its original recipe was identified in some existent museum specimens^2, 8^, the synthetic procedure leading to this particular mixture of compounds is yet to be determined. Perkin have reported in latter investigations, that aniline was contaminated with *orto*- and *para*-toluidine, which then became part of the recipe for tuning the red and blue shade of Mauve^14^: “By using aniline containing much larger quantities of toluidine a redder colouring matter was obtained. By taking advantage of this two different products were manufactured, namely a blue shade of mauve prepared from aniline containing but little toluidine, and a red shade from aniline containing large quantities of toluidine”. It is known that both Perkin and Caro knew how to make red and blue shade mauve (here seen as the colour), according to the compositions, or at least boiling points, of the mixed starting aromatic hydrocarbons (aniline, o-toluidine, p-toluidine). However, there was poor knowledge at the time of the final composition and it is unlikely that the shades of mauve were obtained by synthetic procedures other than those using the above-mentioned aromatic hydrocarbons as starting materials. A possible control of the shade of mauve can lie in the purification step, i.e., to remove other mauveine isomers or to tune the C_26_/C_27_ ratio to improve the shade.

While attempting to reproduce the original Perkin recipe for mauveine, Cliffe^13^ proposed a mixture of aniline (2 mol.) and *o-* and *p-*toluidines (1 mol. each) while others^15^ suggested in the proportion of *p*-toluidine (1.0 equiv.) and aniline and *o-*toluidine (1.5 equi. each). As will be shown these are different to the one we have found to be identified as the one leading to the samples present in the historical samples (ScM1 and CM). Indeed, In the development of our studies towards establishing the exact nature of the dye produced by Perkin^2^, different proportions of aniline, *p-*toluidine and *o-*toluidine were combined for reproducing the pioneer synthesis of mauveine. Two starting compositions of 1:10:10 (MV6) and 1:10:5 (MV5) of aniline, p-toluidine and o-toluidine, were selected for the synthesis of mauveine based on the assumption that larger quantities of toluidines will produce a redder colouring matter with the C_26_ and C_25_ and C_24_ structures emerging as the major mauveine tracers, see Tables 1 and 2. Other proportions including those of 1:4:2 (MV3), 1:4:1 (MV2), 1:2:1 (MV1) and 0:1:0 (MV4) mixtures of aniline with smaller quantities of both toluidines were also tested for producing a more blue-based colouring matter in which C_26_ and C_27_ structures are dominant tracers. A comparative HPLC analysis of the synthesized products, together with a set of historical mauveine samples, obtained from museum collections, was carried out and the complex composition of Perkin’s mauveine was confirmed. In agreement with previous findings^2, 8^ mauveines A (C_26_) and B (C_27_) have emerged as dominant components, while mauveines C_25_ and C_27_ B2 isomers were identified as important tracers of the original synthetic procedure. HPLC plays an important role in analysis of historical organic colorants^16^. Therefore, the analysis (and characterization) of the resulting products was made by HPLC-DAD, followed by a multivariate data analysis procedure based on HCA and PCA. These unsupervised chemometric methods have provided some clear-cut patterns from the HPLC data, and have validated the synthetic route that was likely to have been followed by Perkin.

**Table 1:** Relative percentages of the main chromophores isolated from different mauveine samples at λ = 550 nm, including the ratio between the sum of all C_26_ (mauveine A) and all C_27_ (mauveines B, B2, B3 and B4) compound percentages.

	C_24_	C_25_ab_	C_26_	C_27_	C_27_	C_27_	C_28_	C_28_	C_29_	C_26_/C_27_
				B3 + B4	B2	B	C1			
ScM1	0.3	2.9	28.9	0.0	11.3	18.1	18.6	15.7	4.2	1.0
ScM2	0.1	12.8	8.8	32.4	9.9	8.4	10.1	17.5	0.0	0.2
ScM3	0.0	1.2	34.2	24.0	24.2	9.3	0.0	3.7	3.4	0.6
ScM4	0.0	8.3	26.4	0.0	10.8	24.7	15.5	14.3	0.0	0.7
MS1M1	0.0	3.1	13.4	26.8	6.4	14.7	14.7	11.5	9.4	0.3
MS1M2	49.9	31.3	14.9	0.0	3.9	0.0	0.0	0.0	0.0	3.8
CM	0.4	2.4	28.5	14.4	8.7	17.2	15.6	12.8	0.0	0.7
MV1^a^	0.3	2.0	27.4	13.7	8.3	16.7	14.9	12.2	4.5	0.7
MV2^b^	9.1	24.5	19.2	17.0	30.2	0.0	0.0	0.0	0.0	0.4
MV3^c^	5.3	28.8	26.7	0.0	39.2	0.0	0.0	0.0	0.0	0.7
MV4^d^	5.6	28.3	27.1	0.0	39.0	0.0	0.0	0.0	0.0	0.7
MV5^e^	20.3	38.9	15.7	13.7	11.4	0.0	0.0	0.0	0.0	0.6
MV6^f^	15.6	33.7	26.7	0.0	24.0	0.0	0.0	0.0	0.0	1.1

^a^1:2:1; ^b^1:4:1, ^c^1:4:2, ^d^0:1:0, ^e^1:10:5 and ^f^1:10:10 mole ratio of aniline, *p*-toluidine and *o-*toluidine respectively.

**Table 2:** Retention times (minutes) for the main chromophores of mauveine obtained from different historical and synthesized samples (MV1-6), at λ = 550 nm.

	C_24_	C_25_ab_	C_26_	C_27_	C_27_	C_27_	C_28_	C_28_	C_29_
				B3 + B4	B2	B	C1		
ScM1	23.7	24.8;25.3	26.0	26.6;−	27.2	28.6	29.4	30.0	31.1
ScM2	23.8	24.6;25.3	26.1	26.7;27.7	27.1	28.7	29.4	30.0	—
ScM3	0.0	24.7;25.4	26.2	26.8;28.0	27.2	28.7	—	30.0	31.0
ScM4	0.0	24.8;25.3	26.0	−;27.7	27.2	28.8	29.5	30.1	—
MS1M1	23.8	24.6;25.3	26.0	26.5;27.7	27.2	28.7	29.5	30.1	30.9
MS1M2	23.4	25.0	26.5	—	27.6	—	—	—	—
CM	23.8	24.8;25.3	26.0	26.9;−	27.2	28.8	29.5	30.1	—
MV1	23.8	24.8;25.3	26.0	26.5;−	27.2	28.8	29.5	30.1	31.2
MV2	23.9	24.4;25.1	26.2	26.5;−	27.4	—	—	—	—
MV3	23.9	24.6;24.9	26.1	—	26.9	—	—	—	—
MV4	23.8	24.5;24.9	26.0	—	26.8	—	—	—	—
MV5	24.0	24.8;25.7	26.4	26.8;28.0	27.6	—	—	—	—
MV6	23.8	24.6;25.0	26.1	—	26.9	—	—	—	—

**Figure 2:**
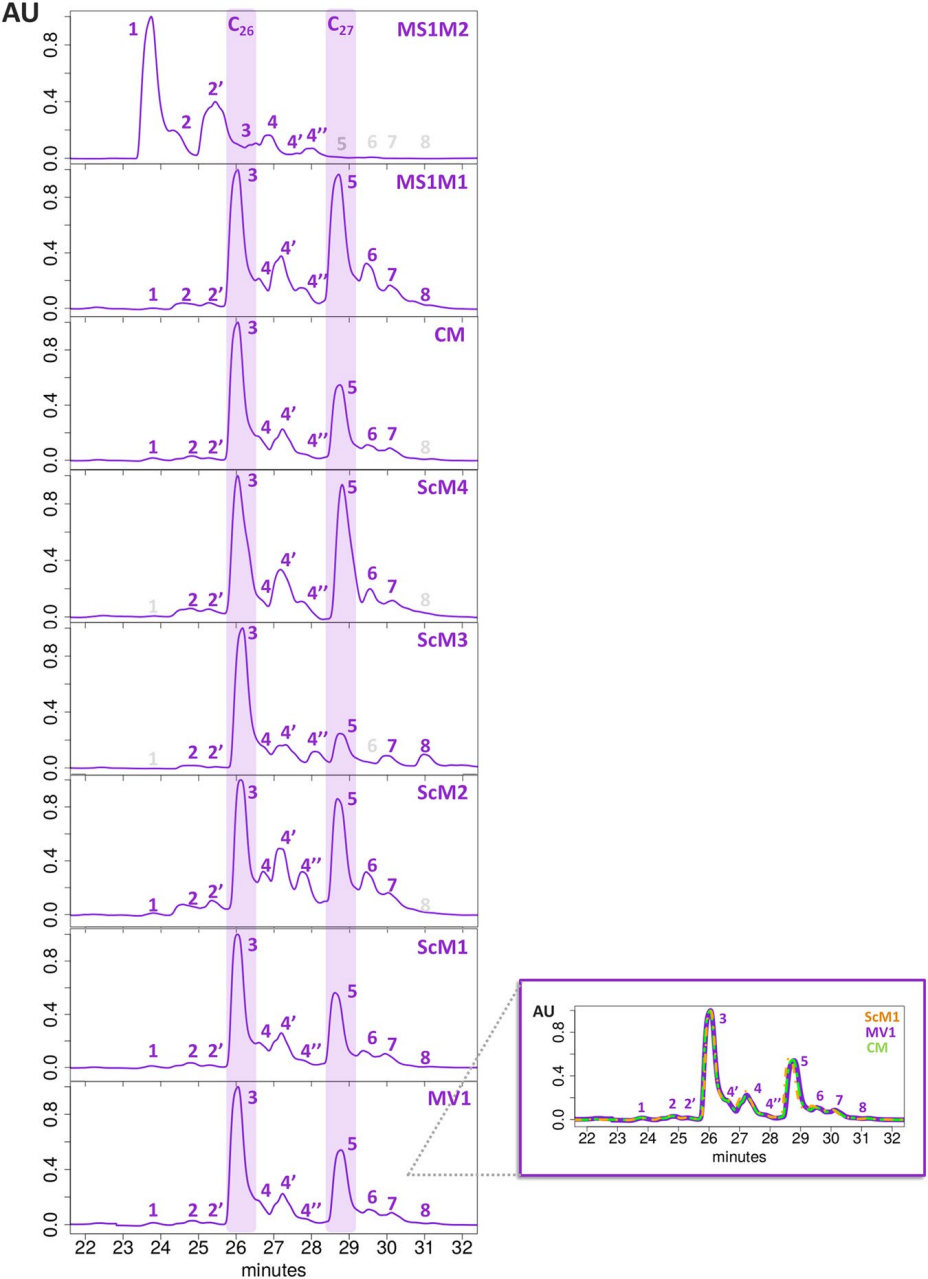
HPLC-DAD chromatograms of historical samples of mauveine salts at λ = 550 nm for Science Museum 1 (ScM1), Science Museum 2 (ScM2), Science Museum 3 (ScM3), Science Museum 4 (ScM4), Museum SI Manchester 1 (MS1M1), Museum SI Manchester 2 (MS1M2) and Chandler Museum (CM). All samples were dissolved in methanol. The major components identified are labelled as: 1–pseudo-mauveine (C_24_); 2 and 2′ – two monomethylated C_25_ isomers (C_25a_ and C_25b_); 3 – one dimethylated mauveine (C_26_); 4, 4′, 4″ and 5 – four trimethylated mauveines (including C_27_ and B2, B3 and B4 isomers); 6 and 7 – two tretamethylated mauveines (C_28_ C, C1 isomers); 8 – one pentamethylated mauveine (C_29_). For details see refs. 2, 8. Purple labels refer to the mauveine structures identified in each sample; grey labels refer to unidentified compounds. In order to illustrate the correct alignment of the peaks between the samples and considering three of the most similar profiles, ScM1, MV1 and CM were represented in the same graph.

The HPLC-DAD chromatograms of mauveine samples obtained at λ = 550 nm are depicted in Fig. 2.

Observation of the chromatograms indicates a broad range of mauveine chromophores with the main structures corresponding to C_24_ (pseudo mauveine), C_25_, C_26_ (mauveine A), C_27_ (mauveine B), C_28_ (mauveine C) and C_29_ (mauveine D). The C_25_ and C_27_ isomers are also identified and are clearly less prevalent. The labelling of the peaks corresponding to the mauveine structures with different number of carbons was based on the comparison of the retention times and the relative peak areas with previous data^2^. From the observation of these profiles, it can be seen that one of the synthesized mauveine, identified as MV1 together with the historical samples are complex mixtures of at least 10 different compounds, all of which containing the 7-amino-5-phenyl-3-(phenylamino)-phenazin-5-ium core. In the sample of the Museum of Science and Industry of Manchester 2 (MS1M2), pseudo-mauveine (C_24_), monomethylated C_25ab_ isomers and dimethylated mauveine A (C_26_) were found as the major components. C_24_ and C_25_ isomers are present as minor tracers in the remaining historical samples. The profiles identified for Science Museum 2 (ScM2), Science Museum 3 (ScM3) and Museum SI Manchester 1 (MS1M1) are in agreement with the previously published analysis^2, 8^. As can be seen from Fig. 2 mauveines A and B are the major components of the mauveine mixture. A clear pattern can be observed: the distribution of the different chromophores in MV1 (Fig. 2) corresponds to the profile of Science Museum 1 and Chandlers Museum (ScM1 and CM respectively) samples. Mauveines A and B are clearly dominant, corresponding to a C_26_/C_27_ ratio approximately equal to 1 (see Table 1). Tables 1 and 2 summarize information on the relative percentage and retention times of the main mauveine chromophores isolated from the synthesized and historical collections, at λ = 550 nm. The ratio between the sum of all C_26_ (mauveine A) and all C_27_ (mauveines B, B2, B3 and B4) compound percentages is also included.

Although the distribution of mauveine compounds amongst the different samples (Table 1) shows significant dissimilarities, the two major chromophores are (with the exception of MS1M2) mauveines A (C_26_) and B (C_27_). Other C_27_ isomers, mauveines B3 and B4, as well as C_28_ mauveines, C and C1, are also present, with mauveine B2 being the most prevalent of these minor compounds. In ScM3, CM, MV1 and ScM1, mauveine A is present in a larger amount, and contributes with ca. 30% for the overall chromophore content. In contrast, when the C_26_/C_27_ ratio is considered, in ScM2 and MS1M1, the major chromophores are the C_27_ isomers, which is in agreement with previously results^2^.

**Figure 3:**
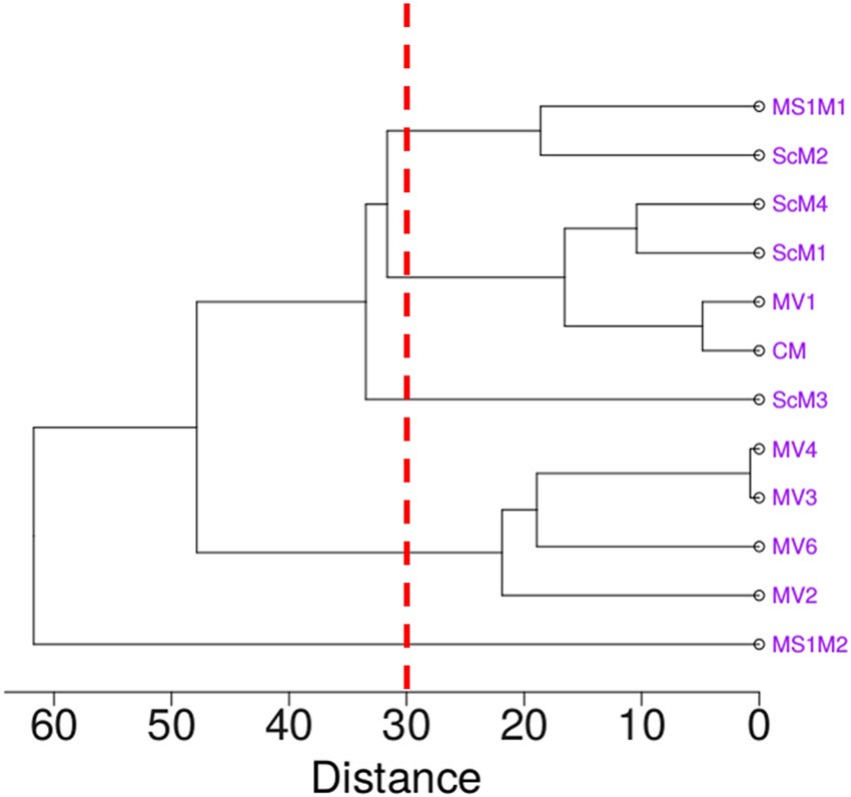
Dendogram obtained for the synthesized samples (MV1-6) and historical samples (ScM1-4, MS1M1-2, and CM) in terms of the distribution of the different chromophores. The construction resorts to the average linkage method with Euclidean distances, using the main chromophores attributed on the basis of their relative integrated areas as variables.

Fig. 3 presents a dendrogram reflecting the similarity between the synthesized samples and the historical samples, considering the relative percentages of different mauveine compounds identified in Fig. 2. This provides a two-dimensional diagram of the data structure, indicating the merging samples and the merging distances. Five groups of samples are identified at a distance of 30, and reflect the C_26_/C_27_ ratio (sum of all C_26_ and all C_27_ compound percentages). For instance, the group on the top includes the samples MS1M1 and ScM2 with the lowest C_26_/C_27_ ratio (0.3 and 0.2 respectively). ScM1, ScM4, CM and MV1 present a C_26_/C_27_ ratio of 0.7–1.0. MS1M2 differ significantly from the other historical samples as the prevalence of C_27_ structures is very low. This sample presents the highest percentage of non-methylated mauveine (C_24_). The group including MV2-6 samples is also isolated as their profiles are quite dissimilar (see Fig. 3 and Tables 1 and 2). These samples contain higher percentages of pseudo-mauveine, mono- and dimethylated mauveines (C_24_, C_25_ isomers and C_26_, respectively), while trimethylated mauveines B, B3 and B4 are minor or even absent compounds.

After establishing the number of clusters, PCA was then directly applied in order to reveal the relationship between the chemical structures in these groups. It is seen that the first two principal components are able to recover more than 72% of the data variability, indicating that a graphical representation based on these two components is clearly meaningful. In fact, the first component contains the most relevant information (ca. 54%) for discrimination, suggesting a one-factor solution.

**Figure 4:**
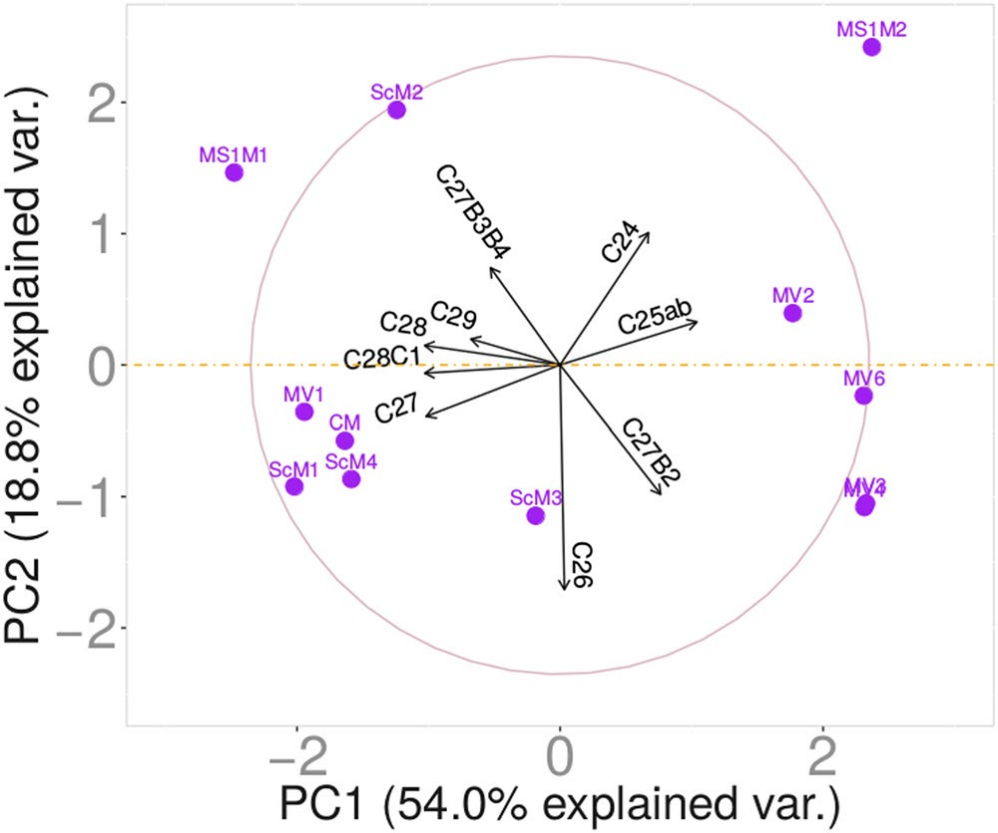
Representation of mauveine samples obtained from the new synthesized and historical collections on the first two components, recovering 72% of variance.

Figure 4 displays a composed view of the samples in a biplot form. The relative position of the samples can be interpreted. Samples that are close together have similar chemical profiles. Both the direction and length of the vectors can also be interpreted. Vectors that point in the same direction correspond to variables that have similar response profiles, and can be interpreted as having similar meaning in the context set by the data. The representation, in two dimensions, allows the visual discrimination between samples. The points representing the mauveine samples are ranked along the first component, PC1, if one takes into account the lower relevance of the second component. PC1 reflects at certain extent the C_26_/C_27_ ratio. Perkin’s mauveine possesses high content of mauveine A and mauveine B (high C_26_/C_27_ ratio) and no fractions of pseudo-mauveine. High values of C_26_/C_27_ and pseudo-mauveine (C_24_) mirror the original synthetic procedure of Caro^8^. ScM1-4, MS1M1, CM and MV1 samples contain mauveine from the process used by Perkin, whereas MS1M2 possesses mauveine obtained using Caro’s procedure. The latter contains the highest content of C_24_ and C_25_ isomers and lower fractions of C_26_ and C_27_. The first component contains essentially information on C_25_ isomers, C_27_ B and B2, C_28_ C and C1 and C_24_ fractions. The second component is mostly related to C_26_, C_24_, and C_27_ isomers B2, B3 and B4. The length of the vectors in Fig. 4 approximates the variances of the ﻿chromophores content. The longer the vector, the higher is the variance. A strong relationship between the C_27_ B, C_28_ C and C1 isomers and C_29_, and also between C_24_ and the C_25_ isomers is observed. Additionally, a weak relationship between C_24_, C_26_ and the C_27_ isomers is confirmed. With one exception (for the C_25_ isomers) the correlation between C_24_ and each of the other mauveine chromophores is negative. MS1M2 stands out through possessing the highest content of pseudo-mauveine and the absence of mauveine A and B.

Other studies^17, 18^ have proposed different starting materials for the synthesis of Perkin’s mauveine; however, based on our approach and comparing with the historical samples (ScM1, and CM), a simple mixture of impure aniline with *orto-* and *para-*toluidine in the (in)correct proportions leads to what can be undoubtedly considered Perkin’s original recipe. The interpretation of the mauveine traces in terms of fragmentation and chromatogram data must be complemented having in mind the relative proportions of the starting materials and the relative combination of specific mauveine isomers (B2, B3, B4 and C1).

In conclusion, we have established a procedure aiming at establishing the (historical) synthesis of mauveine. A simple combination of aniline together with *o*- and *p*-toluidine leads, in the correct proportion, to a mixture of the complex chemical profile of Perkin’s mauve, containing a mixture of several methyl derivatives with a 7-amino-5-phenyl-3-(phenylamino)phenazin-5-ium core. A clear pattern was identified in which mauveines A (C_26_) and B (C_27_) were dominant, while mauveines C_25_ and C_27_ (the B2 isomer) were found to be important tracers to probe the original synthesis. The complementarity between the HPLC-DAD analysis and the unsupervised characterization of the respective data was explored and allowed to determine the synthetic procedure. The inspection of the most discriminating chromophores provides a detailed understanding of the chemical profile. The biplot analysis contains a significant amount of information and can be helpful in interpreting relationships between unknown samples and mauveine fingerprints. It is believed that this approach proves a general route for exploring the chemical profiles for a variety of systems.

## Methods

### Synthesis of Mauveine

The general synthetic procedure used for mauveine consisted of mixing *p*-toluidine (1.14 mmol, 122 mg), Mili-Q water (2.3 mL) and sulfuric acid (0.6 mL, 2.0 M) in a 25 mL glass vial^13^. The reaction vial was magnetically stirred, and an ultrasound-enhanced dissolution was carried out. The reaction vial was then heated gently using a controlled water bath until the reactants dissolved. After the complete dissolution of *p*-toluidine, aniline (0.57 mmol, 0.052 mL), *o*-toluidine (0.55 mmol, 0.06 mL) and 30 mg of potassium dichromate (K_2_Cr_2_O_7_) were added to 160 mL of water. Soon after the addition of K_2_Cr_2_O_7_, the solution turned purple. This describes the synthesis for mauveine MV1, prepared using a 1:2:1 (mole) ratio of aniline, *p*-toluidine and *o*-toluidine, respectively. The other samples followed the same synthetic method, with different proportions of the starting materials and are labelled as MV2-6: 1:4:1, 1:4:2, 0:1:0, 1:10:5 and 1:10:10, respectively. The reaction mixture was stirred and heated at controlled temperature (water bath) for two hours. At the end of the reaction time, the liquid portion was discarded. The resulting dark solid was filtered using gentle suction filtration, and washed with distilled water (slightly heated) until the washing solution becomes colorless. The remaining solid was dried in an oven at ~110 °C for 30 min. It was then washed with petroleum ether until the washings became colorless and dried again for 10 min at 110 °C. Finally, the remaining solid was washed with a 25% methanol/water solution until the solution turned colorless. This aqueous/alcoholic solution was further evaporated. After complete evaporation, 0.3 mL of methanol (100%) was added to the remaining solid and the sample transferred with a filter pipet to a 5 mL vial. The liquid was carefully evaporated until its volume reduced to ca. 0.03 mL. This final methanol solution contains the ultimate product, mauveine (in ca. 2 mg yield).

### Historical samples

The historical samples studied in this work, obtained from the Science Museum in London, Chandler Museum in New York, Museum of Science and Industry in Manchester and Perth Museum in Scotland, were previously analyzed, as described in ref. 2. and compared to the published results related to the distribution of the different chromophores amongst the historical samples.

### HPLC–DAD characterization of the mauveine samples

The distribution of the several mauveine chromophores was established in an analytical Elite Lachrom HPLC-DAD system with L-2455 Diode Array Detector, L-23000 Column Oven (RP-18 end capped column), L-2130 Pump and a L-2200 Auto Sampler. All the samples were dissolved in methanol. A solvent gradient method was performed with methanol (B) and acidic water (C), with a flow rate of 1.5 mL/min for the chromophore separation: 0–8 min; 15% B/85% C; 8–15 min: 50% B/50% C; 15–20 min: 60% B/40% C; 20–25: 70% B/30% C; 25–30 min: 75% B/25% C; 30–40 min: 90% B/10% C; 40–50 min: 90% B/10% C; 50–60 min: 50% B/50% C; 60–70 min: 15% B/85% C. The HPLC-DAD chromatograms of mauveine were acquired at 550 nm.

The algorithm for baseline correction and peak identification in chromatograms was developed by the authors using R version 3.3.1^19^ and the Alternating Least Squares (ALS) for the Automatic Chemical Exploration of mixtures package^20–22^. This has been implemented and validated for use in data from systems like HPLC-DAD^23^. Baseline estimation was built on the Asymmetric Least Squares^23^, a method in which a second derivative constrained weighted regression algorithm is used for baseline correction. This was performed using a second derivative constraint of 10, a weighting of positive residuals parameter of 0.01, and a maximum of 20 iterations. For resolved peaks and for peaks in regions with an approximately linear baseline this method gives a good estimated of the baseline. However, for unresolved peaks, the first and second derivatives of the original profiles allowed to identify less marked features. Zeros in the first derivative, from positive values, were assigned to definite maxima. Inflection points and inverted peaks given by the second derivative were used for confirmation. As the number of mauveine compounds was previously identified^2, 8, 9^, a method based on fitting chromatographic peaks with a Gaussian profile was used for determining the fit peak parameters. For peak grouping, it was assumed that peaks in chromatograms having similar retention time originate from the same chemical compound. No further corrections were made for determining the sample to sample variation of the retention times, as no significant shifts were identified between the historical and the new synthesized samples.

### Multivariate data analysis procedure

The analysis resorts mainly to methods that involve the simultaneous study of several key variables related to the different mauveine chromophores. In this context, multivariate methods allow a detailed exploration into possible patterns, enable inter-relationships between the different chromophores to be represented graphically, and provide ways of simplifying and reducing the dimensionality of the data to predict the proportions of the starting materials, in order to reproduce the historical synthesis of mauveine. The main goal is to provide an interpretation of the different chemical profiles, taking simultaneous account of several mauveine compounds. A similar analysis was performed in a previous work related to specific mauveine profiles found in UK Victorian postage stamps, including a comparison with historical samples from both Perkin’s and Caro’s recipes. A selection of more than 35 postage stamps was analyzed, covering the period of 1847–1901^8^, to identify the use of mauveine as a dye for those stamps and characterize the corresponding synthesis. The chemical profile was established and classified using standard chemometrics methods, according to the main colorants of the historical mauveine samples, attributed on the basis of their m/z and respective fragmentation patterns. The characterization of mauveine samples was conducted resorting to two well established methods: (i) hierarchical cluster analysis (HCA) for defining the data structure and (ii) principal component analysis (PCA) for data overview and feature selection. HCA is especially suitable for cases in which there is no *a priori* identification of classes, suggesting a structure of the data based on clusters. These clusters are further detailed and rationalized by PCA with biplot representation. The latter techniques allow the visualization of the data, and, thus, lead directly to observation of the most relevant patterns.

The procedure based on HCA and PCA requires a description of the objects, i.e. points in Euclidean space. In this analysis, each mauveine sample corresponds to one of these points. Both sets (mauveine MV1-6 and historical samples) are described on the basis the components related to the mauveine structures (C_24_, two C_25_ isomers–C_25ab_, C_26_–mauveine A, four C_27_–mauveine B–isomers (C_27_, C_27_B2 and C_27_B3 + B4), C_28_–mauveine C–and the C1 isomer, and C_29_–mauveine D. The data set contains information on 13 analyzed samples (6 synthesized samples, 7 historical samples obtained from the Science Museum, London, UK, (ScM1-4), the Museum of Science and Industry, Manchester, UK, (MS1M1-2) and the Chandler Museum, Arizona, USA, (CM). More details on the data normalization, data structure and dimensionality reduction procedure can be found in the Electronic Supplementary information section.

## Electronic supplementary material

Supplementary Information
