# Supplementary material for: Reconstructing the historical synthesis of mauveine from Perkin and Caro: procedure and details
Source: Sci Rep. 2017 Jul 28;7:6806. doi: 10.1038/s41598-017-07239-z (PMC5533699; doi:10.1038/s41598-017-07239-z)
Supplement: Supplementary file 1 — Supplementary Information [file 41598_2017_7239_MOESM1_ESM.doc]

Electronic Supporting Information

Reconstructing the historical synthesis of mauveine from Perkin and Caro: procedure and details

Tânia F. G. G. Cova, Alberto A.C.C. Pais and J. Sérgio Seixas de Melo*

Coimbra Chemistry Centre, Department of Chemistry, University of Coimbra, P3004-535 Coimbra, Portugal

* sseixas@ci.uc.pt

Table of Contents for Electronic Supporting Information

**Fig.S1.** Similarity among the UK Victorian postage stamps and the historical samples in terms of the distribution of the different chromophores. Dendrogram constructed resorting to the average linkage method with Euclidean distances, using the main chromophores attributed on the basis of their m/z and respective fragmentation patterns as variables defining each sample.

**Fig. S2.** Representation of the UK Victorian postage stamps and the historical samples on the first two components, recovering 66.5% of variance.

### **Data normalization**

### **Setting of the data structure**

### **Dimensionality reduction procedure**

**
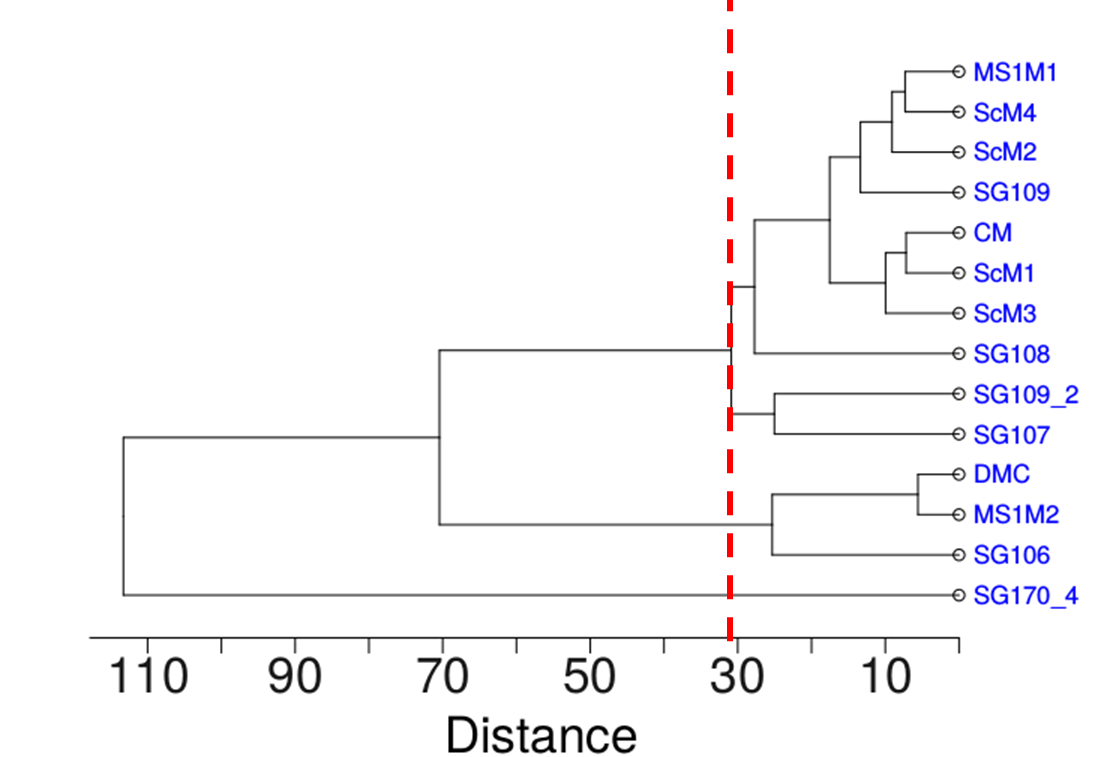
**

**Figure S1.** Similarity among the UK Victorian postage stamps and the historical samples in terms of the distribution of the different chromophores. Dendrogram constructed resorting to the average linkage method with Euclidean distances, using the main chromophores attributed on the basis of their m/z and respective fragmentation patterns as variables defining each sample.

**
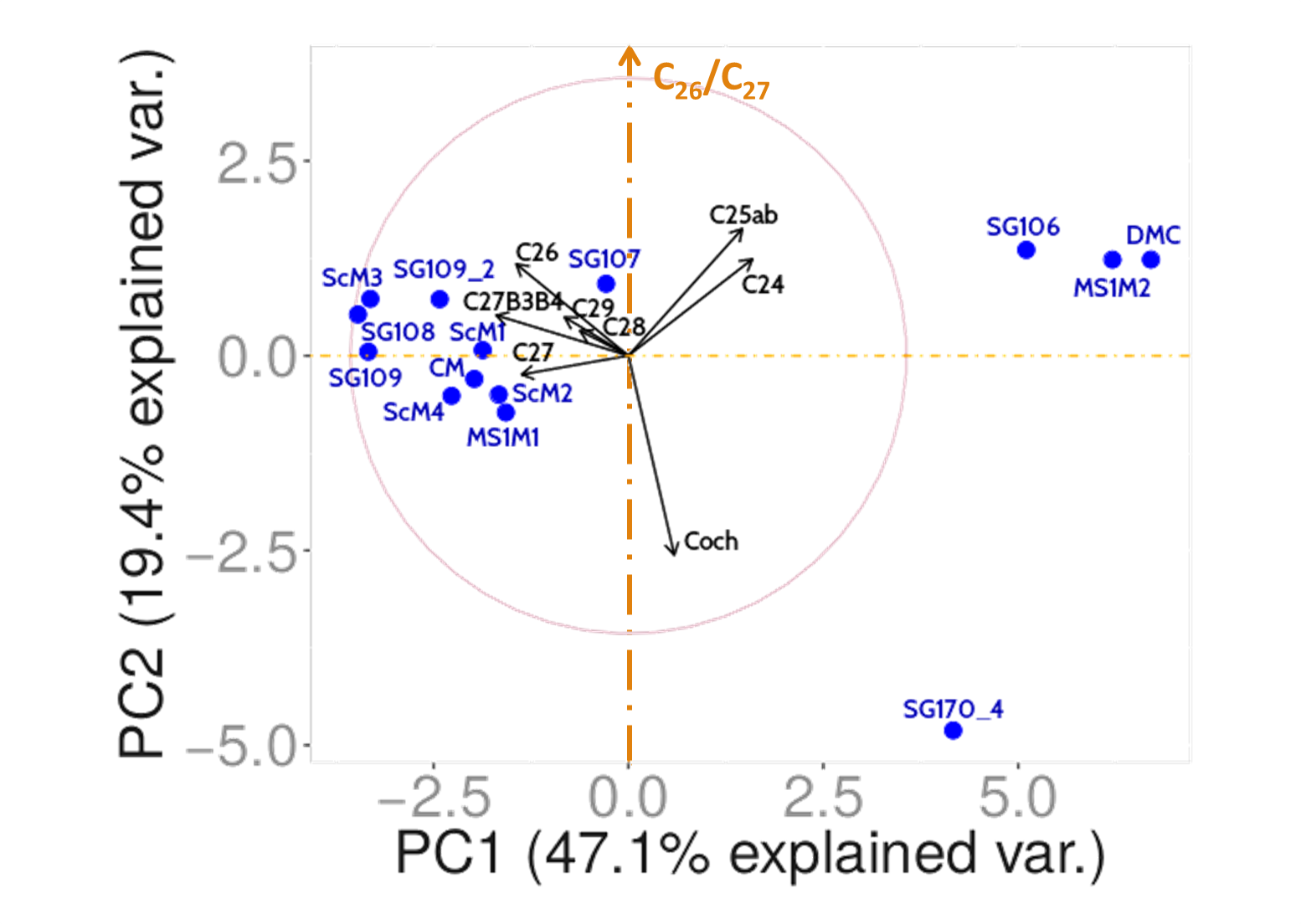
**

**Fig. S2.** Representation of the UK Victorian postage stamps and the historical samples on the first two components, recovering 66.5% of variance.

### **Data normalization**

A fundamental question concerns the normalization of the data. In this case, each variable is used in the form of a fraction
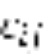


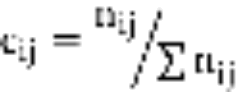
 (1)

pertaining to chromophores of type i in sample j; *n_ij_* the relative percentage determined from the integrated area of the peak related to that chromophore in that sample, and
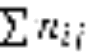
 the total area or retention time for each sample. This simply means that the area (or retention time) for each chromophore is divided by the total amount for that same sample. As such, each sample is described by a set of variables, which consist on the fraction of amounts for each type.

### **Setting of the data structure**

The main goal of clustering algorithms is to group the data into a number of sensible clusters according to their similarities. In the exploratory data analysis, Hierarchical Cluster Analysis (HCA) is used. A recent review of this can be found in Ref. 1.^1^ The use of hierarchical methods is extremely common, and one of the reasons is that they allow the visualization of the data structure, even in complex cases. The HCA procedure is graphically represented by a dendrogram, which consists of a cluster structure and illustrates the fusions, or divisions, made at each successive stage of the analysis. It allows inspection of the overall structure of the data, and estimation of the number of clusters.

Mauveine samples with similar chemical profiles are linked, based on the respective composition. The result of the association is the hierarchical structure, in which similar samples are merged.

The Euclidean distance,


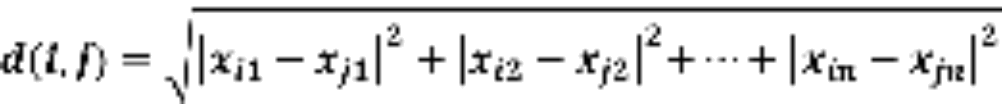
 (2)

is the most popular way to determine the similarity between samples (i and j).

The average linkage method is a standard linkage procedure, and has been selected for the present study. In this method the distance between two groups of samples is established on the basis of the average distance between samples in one cluster and samples in the other cluster. At each stage of the process, the smallest average linkage distance is selected for combining the clusters. Once the similarity measure and the linkage method are defined, the agglomeration of samples and groups in each step of the process follows the order of larger similarity.

### **Dimensionality reduction procedure**

Principal component analysis (PCA) is one of the simplest and most robust ways of taking high-dimensional data, and using the dependencies between the variables to represent it in a more tractable, lower-dimensional form, respecting structure and preserving variance ^2, 3^. By construction, the first principal component is the one which maximizes the variance (reflected by its eigenvalue) when data are projected onto a line, which corresponds to a direction in the *p*-space, assuming a number of *p* variables, while the second one is orthogonal to it, and still maximizes the remaining variance. For this reason, using the first two components should yield the better approximation of the original variables space when it is projected onto a plane.

PCA computes a compact and optimal description of the data set, providing a roadmap to a lower dimension space that reveals the underlying structure. The most influential variables in the system are highlighted, and the most relevant factors may be identified. This technique relies on the assumption that most of the information contained on the data is present in directions along which the variations are the largest. In the present work, PCA summarizes the information residing in the data corresponding to the chemical analyses of the set of mauveine samples, into a form which may be more easily inspected and interpreted. The original multi-dimensional space, defined by those chromophores, is contracted into a few descriptive dimensions, which represent the main variation in the data. Each principal component (PC) can be displayed graphically and analyzed separately; its meaning may often be established on the basis of a few chemical features. Essentially, the procedure is carried out by a linear transformation of the *m* chromophores corresponding to different chemical structures *x_i_* into a new set, the principal components *u_i_*


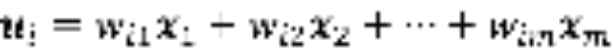
 (3)

where *w_i1_*. . .*w_im_* are the loadings, i.e. the weights of the each chromophore in the linear combination.

Since the first principal components retain most of the variance, several variables can be summarized by a few components and a plot of the first two or three principal components enables the visualization of most of the information contained in the data. PCA requires the solution of an eigenvalue problem, either based on the correlation or variance/covariance matrices of the original variables. In either case, these components are ranked, and the percentage of explained variance *λ_i_* decreases from the first principal component to the second and so on ^2^, suggesting the criteria for the selection of the most relevant first *p* principal components. The most common one is Pearson’s,^2^ which can be used in both the variance/covariance and the correlation approaches. The value *p* is selected as the minimum integer that warrants


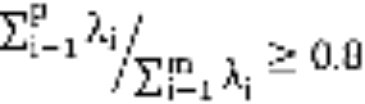
 (4)

If the correlation matrix is used, the most common criterion corresponds to retain the *p* components for which
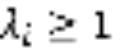
although other values have been suggested.^2^

1. Almeida J, Barbosa L, Pais A, Formosinho S. Improving hierarchical cluster analysis: A new method with outlier detection and automatic clustering. *Chemometrics and Intelligent Laboratory Systems* **87**, 208-217 (2007).

2. Jolliffe I. *Principal component analysis*. Wiley Online Library (2002).

3. Cova TF, Pereira JL, Pais AA. Is standard multivariate analysis sufficient in clinical and epidemiological studies? *Journal of biomedical informatics* **46**, 75-86 (2013).
